# Comparative Study of Lefaxin Family in Two Asian Leeches: *Hirudinaria manillensis* and *Whitmania pigra*

**DOI:** 10.3390/biology14080918

**Published:** 2025-07-23

**Authors:** Tianyu Ye, Fang Zhao, Mingkang Xiao, Jingjing Yin, Rui Ai, Lizhou Tang, Zichao Liu, Zuhao Huang, Gonghua Lin

**Affiliations:** 1 School of Life Sciences, Key Laboratory of Jiangxi Province for Biological Invasion and Biosecurity, Jinggangshan University, Ji’an 343009, China; yetianyu0220@163.com (T.Y.); zf_lgh@163.com (F.Z.); xmk18379773872@163.com (M.X.); m15656142610@163.com (J.Y.); 18008109213@163.com (R.A.); 2 College of Life Sciences, Jiangxi Normal University, Nanchang 330022, China; tanglizhou@163.com; 3 Engineering Research Center for Exploitation and Utilization of Leech Resources in Universities of Yunnan Province, School of Agronomy and Life Sciences, Kunming University, Kunming 650000, China; abclzc@aliyun.com

**Keywords:** medicinal leech, molecular evolution, gene expression, anticoagulant activity, Factor Xa inhibitors

## Abstract

Thrombotic diseases represent a significant global health burden, particularly for middle-aged and elderly populations. Medicinal leeches, such as *Hirudinaria manillensis* and *Whitmania pigra*, have been traditionally used for their anticoagulant properties. The genomes of these leeches each harbor three lefaxin genes, which are designated *lefaxin_Hman1–3* and *lefaxin_Wpig1–3*, respectively. We conducted genomic and transcriptomic sequencing on wild populations of both species. Bioinformatics tools were employed to analyze intraspecific variation, molecular evolution, and protein structures. We expressed recombinant lefaxin proteins in *Pichia pastoris* and assessed their anticoagulant activities using in vitro coagulation assays. *H. manillensis* exhibited greater genetic diversity and stability, whereas *W. pigra* showed higher expression levels and hydrophilicity. Both species exhibited purifying selection, indicating conserved function, and their lefaxin structures are similar to the archetypal lefaxin (UniProt No. P86681.1). *W. pigra* lefaxins bound Factor Xa more effectively. *W. pigra* lefaxins exhibited more robust anticoagulant activity in vitro compared to those from *H. manillensis*. *W. pigra*, a non-hematophagous leech, shows potent anticoagulant activity through lefaxins, challenging traditional views on leech efficacy. This study underscores the potential of lefaxins as therapeutic targets for thrombotic diseases and highlights the need to reconsider the use of various leech species in medicine.

## Simple Summary

The hematophagous buffalo leech (*Hirudinaria manillensis*) and the non-hematophagous medicinal leech (*Whitmania pigra*) have long been utilized in traditional Chinese medicine for treating blood-stasis-related diseases. Despite their widespread use, the underlying pharmacological mechanisms remain insufficiently understood. This study combined next-generation sequencing and recombinant expression to systematically compare the lefaxin family (a Factor Xa inhibitor) between both species. Three lefaxin genes (designated *lefaxin_Hman1–3* and *lefaxin_Wpig1–3*) were identified in each sequenced genome of each species, with a focus on intraspecific variation, molecular evolution, gene expression, and anticoagulant activity. The comparative analysis reveals that lefaxin genes in both leech species are subject to purifying selection, emphasizing their conserved functions and roles in anticoagulation. Lefaxins derived from *W. pigra* demonstrate more vigorous anticoagulant activity compared to those from *H. manillensis*. These findings challenge traditional assumptions regarding the medicinal efficacy of leeches, propose lefaxins as a promising therapeutic target for thrombotic diseases, and underscore the need to reevaluate the medical potential of non-hematophagous leech species.

## 1. Introduction

The incidence, disability rate, and mortality associated with thrombotic diseases have consistently increased, positioning them as a significant global health concern, especially among middle-aged and elderly populations [1]. Thrombi, abnormal intravascular clots that impede circulatory flow, can induce ischemic tissue necrosis and organ dysfunction by reducing blood supply in various pathologies [2]. While current anticoagulants (e.g., heparin, warfarin) remain mainstay therapies, their limitations, including bleeding risks and narrow therapeutic windows, indicate that there must be novel treatments targeting alternative pathways in the coagulation cascade [3]. Leeches (Annelida: Clitellata: Hirudinida) are an ancient therapeutic tool with long-standing medicinal use across many cultures, particularly in traditional bloodletting practices [4]. Recognized in modern complementary and alternative medicine [5,6,7], leeches hold significant value in traditional Chinese medicine (TCM) for their hematological effects. The *Shen-Nong-Ben-Cao-Jing* first documented the use of leeches for treating “blood stasis, amenorrhea, abdominal masses, and fluid retention” [8]. Leeches exhibit diverse pharmacological effects, including anticoagulation, vasodilation, and menstrual regulation [9]. Contemporary applications of leeches focus on cardiovascular and cerebrovascular diseases, as well as thrombotic disorders, through their blood-activating and stasis-resolving properties [10].

Leeches exhibit remarkable species diversity. Those commonly employed in TCM include *Hirudo nipponia*, *Whitmania pigra*, *Whitmania acranulata*, and the Asian buffalo leech (*Hirudinaria manillensis*) [11]. Of these, *H. nipponia*, *W. pigra*, and *W. acranulata* are officially listed in the *Chinese Pharmacopoeia* [12]. Although *H. manillensis* is not included in the *Chinese Pharmacopoeia*, its medicinal value has been recognized and utilized regionally, with documented inclusion in the local standards for Chinese medicinal materials in Guangxi [13] and Yunnan provinces [14].

Leeches can be broadly classified, based on their feeding habits, into hematophagous (blood-feeding) and non-hematophagous types [15]. *H. manillensis* predominantly parasitizes humans, domestic animals, and other vertebrates [16,17]. This species has a wide distribution across South and Southeast Asia, as well as southern China, primarily inhabiting lowland ecosystems, including rice paddies, swamps, and ponds [18]. In contrast, *W. pigra* represents a non-hematophagous species that feeds on mollusk hemolymph, particularly from snails and clams [19]. The species *W. pigra* is primarily distributed across most regions of China and certain areas of Japan [20].

*H. manillensis* and *W. pigra* are the dominant species in the Chinese medicinal leech market, being the most frequently used for medicinal purposes [21,22]. By comparison, the smaller size and slower reproduction rate of *H. nipponia* result in reduced aquaculture profitability, with current populations inadequate for market requirements [23]. Although studies demonstrate the superior antithrombotic efficacy of *H. manillensis* over both *H. nipponia* and some non-hematophagous leeches [24], such findings should not diminish the research potential of non-hematophagous species. Processed leeches such as *W. pigra* have maintained historical significance in medicinal applications [25]. While thermal processing often denatures polypeptides and protein-based components, such as hirudin, *W. pigra* exhibits enhanced anticoagulant activity post-decoction, contrasting with *H. manillensis*, which shows reduced anticoagulant capacity after heat treatment [26]. These differential responses highlight the distinct therapeutic merits of non-hematophagous leeches that merit comprehensive investigation.

Recent advances in modern medicine have identified a diverse array of antithrombotic proteins and peptides in leeches, with hirudin being the most prominent [27]. Hirudin consists of approximately 65 amino acids and has a molecular weight of around 7000 Daltons [28]. It functions as a potent natural thrombin inhibitor. The high specificity of hirudin for thrombin enables it to effectively inhibit the conversion of fibrinogen to fibrin catalyzed by thrombin [29]. In addition to hirudin, other bioactive substances have been identified in leeches, including antistasin [30], a Factor Xa inhibitor; decorsin [31], a platelet aggregation inhibitor; and bdellastasin [32], an anticoagulant protein. These substances are capable of exerting anticoagulant effects either directly or indirectly.

Factor Xa, a vitamin-K-dependent coagulation protease, catalyzes thrombin generation from prothrombin in the presence of Factor Va, calcium, and phospholipids [33]. This central role in coagulation cascade activation has positioned Factor Xa as a key therapeutic target for anticoagulant development, particularly in managing thrombotic disorders such as stroke, myocardial infarction, and deep vein thrombosis [34]. Lefaxin, a 30 kDa Factor Xa inhibitor (isoelectric point~5.7) isolated from *Haementeria depressa* salivary glands, demonstrates significant anticoagulant potential by specifically inhibiting Factor Xa activity within the prothrombinase complex [35].

Studies have demonstrated that the anticoagulant activities of leech genes may differ significantly even within the same species. For example, while multiple hirudin genes exist in the *H. manillensis* genome, only select variants demonstrate anticoagulant properties [36]. This functional divergence suggests that structural or sequence variations in amino acid composition may influence hirudin efficacy [37]. Interspecies genetic variations further contribute to the differential composition, functionality, and evolutionary patterns of antithrombotic genes across leech species, where homologous genes might be expressed in one species but silenced in another through genomic absence or regulatory differences [21].

Our previous research has identified at least 72 antithrombotic genes within the genome of *H. manillensis* [37], spanning 21 gene families. The lefaxin genes in *H. manillensis* comprise three members (*lefaxin_Hman1–3*). The genome of *W. pigra* contains at least 79 antithrombotic genes distributed across 20 gene families [38], and the lefaxin genes comprise three members (*lefaxin_Wpig1–3*). This study aims to investigate whether the lefaxin genes exhibit antithrombotic activity in these two leech species through bioinformatics analysis, gene expression profiling, and in vitro anticoagulant activity assays and to compare the similarities and differences between the lefaxins of *H. manillensis* and *W. pigra*.

## 2. Materials and Methods

### 2.1. DNA and RNA Sequencing

We sampled wild populations of *H. manillensis* and *W. pigra* from various locations. Specimens of *H. manillensis* were collected from Yulin, Guangxi (GXYL, 110.57° E, 22.81° N); Zhanjiang, Guangdong (GDZJ, 110.46° E, 21.25° N); and Honghe, Yunnan (YNHH, 102.58° E, 23.31° N). We also collected *W. pigra* specimens from Baodi, Tianjin (TJBD, 117.48° E, 39.47° N); Yibin, Sichuan (SCYB, 105.31° E, 28.18° N); and Wuhan, Hubei (HBWH, 114.57° E, 30.59° N). After a two-week acclimatization period under laboratory conditions (temperature maintained at 25 ± 1 °C, 70 ± 5% relative humidity, 12 h light/dark cycle, and daily water renewal), 10 adult individuals were randomly selected from each of the collection sites. The anterior one-third of the head tissue was excised from each specimen, and any residual blood was washed away using sterile water. We extracted total DNA using the DNeasy Blood and Tissue Kit (Qiagen, Germany). We checked the quality and condition of the DNA using agarose gel electrophoresis, NanoDrop spectrophotometry (NanoDrop Technologies, Wilmington, DE, USA), and Qubit fluorometry (Thermo Fisher Scientific, Waltham, MA, USA). High-quality genomic DNA was then used to construct sequencing libraries for the PacBio and Illumina platforms.

For RNA-Seq, total RNA was extracted from individual leeches using TRIzol reagent (Thermo Fisher Scientific Inc.) and purified with the RNeasy Mini Kit (Qiagen, Chatsworth, CA, USA). We constructed and sequenced a 350 bp insert DNA library on the Illumina HiSeq 2000 platform using paired-end 150 bp reads. We processed the raw sequences using fastp v0.20.0 [39] to remove adapters and low-quality regions, yielding clean reads for further analysis.

### 2.2. Sequence Extraction

We performed de novo assembly of genome resequencing clean reads using MEGAHIT v1.2.9 [40], which generated contigs for each sample. For transcriptome data, we assembled clean reads using Trinity v2.9.0 [41] to obtain unigene sequences. We used published lefaxin genes as bait sequences for subsequent analysis. We formatted the unigene sequences into a BLAST database and identified homologous sequences using BLAST v2.13.0+ [42].

Using MEGA v11.0.13 [43], we aligned the homologous sequences and examined variant sites. Based on the alignment results and intron boundary information (GT-AG rule), we extracted coding regions. For genes with low expression levels that lacked complete coding sequences, we used exons ±50 bp as bait to retrieve homologous contigs through BLAST searches, followed by alignment in MEGA. To resolve highly variable regions that likely resulted from assembly gaps, we employed Mirabait [44] to extract and align related reads, thereby improving sequence accuracy.

### 2.3. Intraspecific Variation Analysis

We compiled the coding region sequences of each gene into FASTA format and aligned them using the “Align by MUSCLE (Codons)” function in MEGA. We then determined the number of variable sites (VS) and number of haplotypes (HN) for each gene using DnaSP [45]. VS reflects mutation frequency within gene sequences, while HN indicates genetic diversity. We calculated Watterson’s Theta diversity (WD) index, which measures genetic variation within populations, using DAMBE v7.3.5 [46]. For further analysis, we changed gene sequences into amino acid sequences in MEGA and then calculated VS, HN, and WD for these sequences using DAMBE.

### 2.4. Molecular Evolution Analysis

Lefaxin genes from four leech species (*H. manillensis*, *W. pigra*, *H. nipponia*, and *Hirudo medicinalis*) were aligned, and a phylogenetic tree was constructed using MEGA. Based on the relationships among the four species [47], a species tree of these lefaxins was built using TreeGraph v2.15 [48]. The *dN/dS* (*ω*) ratios were estimated using PAML-X [49] to evaluate the selective pressures acting on each lefaxin gene. Synonymous substitutions are assumed to be neutral, whereas nonsynonymous substitutions may be subject to purifying (*ω* < 1), neutral (*ω* = 1), or positive (*ω* > 1) selection.

Three models from the CODEML module of PAML were applied: the branch model, branch-site model, and site model. In the branch and branch-site models, branches of *H. manillensis* and *W. pigra* were set as foreground, with the others as background. The site model analysis employed three models: M0, M7, and M8. By examining the *ω* ratios derived from these models, we elucidated how different types of selective pressures may have influenced various members of the lefaxin family during their evolutionary history.

### 2.5. Gene Expression Analysis

We constructed sequence indices using the Salmon v1.0.0 [50] based on coding region sequences from whole-genome annotation data. We aligned the transcriptome reads from each sample to these indices using a k-mer value of 31, which yielded transcripts per million (TPM) values for each coding region sequence. This information was used to determine and compare the relative expression levels of genes across samples.

We analyzed differences in the levels of lefaxin gene expression among populations within species using SPSS v25 [51]. A one-sample Kolmogorov–Smirnov test indicated that lefaxin expression levels significantly deviated from normality (*p* < 0.05). Given this result, we employed non-parametric methods: first, we used the Several-Related-Samples test (Friedman test) to assess significant differences among genes. If significant differences were detected, we performed pairwise comparisons using the Two-Related-Samples test (Wilcoxon test).

Differences in lefaxin gene expression between species were analyzed using SPSS software. The one-sample Kolmogorov–Smirnov test was used to check for a normal distribution (*p* > 0.05). If the data had a normal distribution and the variances were homogeneous (as determined by Levene’s test, *p* > 0.05), we used an Independent Samples T-test. If not, we applied the non-parametric Mann–Whitney U test.

### 2.6. Analysis of Protein Biochemical Properties

We translated the six lefaxin genes into their corresponding protein sequences. We used SignalP [52], an online tool, to predict signal peptide regions and evaluate the presence of signal peptide domains. Additionally, we used the ProtParam online tool to calculate the isoelectric point (pI), instability index (II), aliphatic index, and grand average of hydropathy (GRAVY) for each protein [53].

### 2.7. Protein Structure Alignment Analysis

We predicted the protein structures of six lefaxins using the AlphaFold Protein Structure Database, along with the archetypal lefaxin (UniProt No. P86681.1). To assess their structural similarity, we employed the FATCAT method [54], which allows flexible protein alignment by detecting hinges and internal rearrangements.

We utilized parameters such as root mean square deviation (RMSD), FATCAT score, and *p*-value during the alignment process to assess structural similarity. These parameters quantitatively measured structural conservation and highlighted specific similarities and differences among lefaxins from different species. This analysis offers insights into their evolutionary and functional relationships.

### 2.8. Protein Docking Analysis

The ZDOCK server [55] docked the archetypal lefaxin and six lefaxins with human Factor Xa, treating Factor Xa as the receptor and lefaxins as ligands. ZDOCK is a protein docking algorithm that predicts protein–protein binding modes through energy minimization. After docking, we used PyMOL 3.1 [56] to visualize protein structures and analyze interaction interfaces. Subsequent analysis of the docking results included calculating the binding free energy and evaluating the characteristics of amino acid residues at the binding sites to assess the stability and biological significance of the docking modes.

### 2.9. Pichia Pastoris Expression

Plasmids containing the six genes were synthesized and ordered from Sangon Biotech (Shanghai, China), retaining the necessary sequences for full gene expression. We cultured *Escherichia coli* in a liquid LB medium overnight with shaking to amplify the plasmid DNA. The plasmid DNA was extracted using a commercial kit, linearized with the SpeedyCut SacI enzyme, and purified using a corresponding purification kit.

The linearized plasmid DNA was chemically transformed into GS115-competent cells, which were then plated on Yeast Extract Peptone Dextrose (YEPD) medium with 0.25% geneticin. Yeast colonies appeared after 3–5 days. The successfully transformed yeast cells underwent stepwise resistance selection on a YEPD medium with increasing concentrations of geneticin (0.5%, 1%, and 2%). We inoculated the selected yeast cells into a buffered glycerol-complex medium for expansion. We extracted yeast genomic DNA using a rapid extraction kit and confirmed the integration of the target gene into the yeast genome by PCR amplification and agarose gel electrophoresis. After confirming successful integration, we transferred the yeast cells to the buffered methanol-complex medium for fermentation, which induces the expression of the target gene. We purified the expressed proteins by salting out and desalting them to obtain the final product. We separated the six recombinant proteins by SDS-PAGE and confirmed the presence of the target protein by comparing the molecular weight of the obtained bands with those of the target bands.

### 2.10. Anticoagulation Test

We evaluated the anticoagulant activity of the target proteins using a Sienco hemorheology analyzer (Viscell, UK), which measures clotting kinetics and platelet function using ultrasonic viscoelastic detection. We prepared 500 μL of citrated pig blood (3.8% sodium citrate) and mixed it with 100 μL of the target protein. To reverse anticoagulation, we added 20 μL of 0.25 M calcium chloride to reinitiate clotting capability. We transferred the mixture into a test cup containing 360 μL of whole blood and analyzed it with the Sonoclot system. The instrument’s ultrasonic sensor (200 Hz oscillation) monitored real-time changes in blood viscosity and elasticity during clot formation, generating a coagulation signal curve.

We tested each protein sample in triplicate and recorded the following parameters: (1) Activated clotting time (ACT): Normal range, 100–240 s; values exceeding 240 s indicate prolonged coagulation. (2) Clot rate (CR): Normal range 10–35; values < 10 suggest impaired clotting. (3) Platelet function (PF): PF is quantitatively derived from the timing and quality of clot retraction, reflecting platelet contractile activity during the late phase of coagulation. Values > 1 indicate normality, while values < 1 indicate bleeding risk. All three parameters were automatically calculated by the Sonoclot system based on viscoelastic changes observed during the clotting process. We inferred anticoagulant activity based on delayed or inhibited clot formation after calcium addition, as evidenced by divergence from control curves. This method allowed us to assess the target protein’s effects on hemostasis quantitatively.

## 3. Results

### 3.1. Intraspecific Variation

In the reference genomes of *H. manillensis* and *W. pigra*, six lefaxin genes were identified and named as *lefaxin_Hman1*–*3* and *lefaxin_Wpig1*–*3*, respectively. The coding sequences and amino acid sequences of these genes, alongside those of *H. nipponia* and *H. medicinalis*, were aligned to enable comparison across species (Supplementary File S1). Complete coding sequences for *lefaxin_Hman1–3* (all 32 *H. manillensis* samples, Supplementary File S2) and *lefaxin_Wpig1–3* (all 35 *W. pigra* samples, Supplementary File S3) were successfully obtained. After translating and aligning the valid sequences to analyze sequence variation, a total of 20 variable sites were identified at the DNA level, and 5 were identified at the protein sequence level. The VS in the lefaxin genes of *H. manillensis* was significantly higher than that in *W. pigra* at both the DNA and protein levels. Additionally, 30 DNA haplotypes and 11 amino acid haplotypes were identified, with *H. manillensis* exhibiting a significantly higher HN at both levels compared to *W. pigra*. The WD index for both the DNA and protein levels also indicated significantly higher diversity in *H. manillensis* (Table 1).

In summary, the lefaxin genes in *H. manillensis* demonstrated greater genetic diversity and more haplotypes at both the nucleotide and amino acid levels. In contrast, although *W. pigra* also exhibited some genetic variation, it had fewer variation sites and haplotypes in the lefaxin genes compared to *H. manillensis*. These findings offered helpful information about the molecular evolutionary processes and ecological adaptive differences between these two leech species.

### 3.2. Molecular Evolution

**Figure 1:**
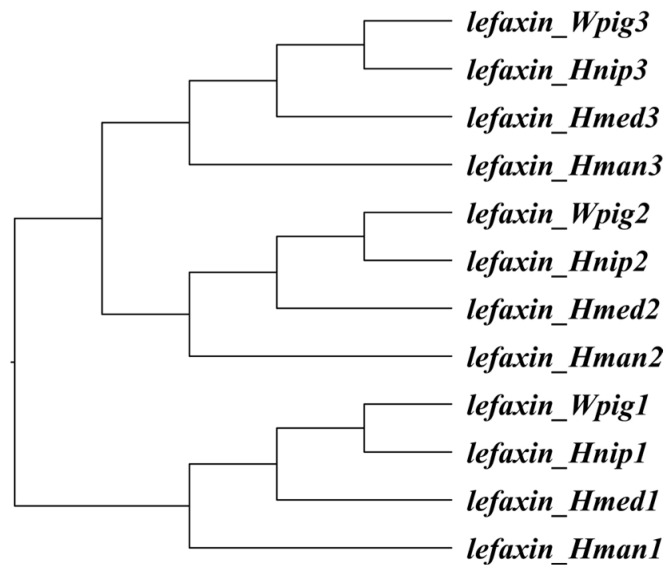
Species tree constructed from lefaxin gene nucleotide sequences of Hirudinaria manillensis (lefaxin_Hman1–3), Whitmania pigra (lefaxin_Wpig1–3), Hirudo nipponia (lefaxin_Hnip1–3), and Hirudo medicinalis (lefaxin_Hmed1–3). Three lefaxin homologs from each species were included to infer their evolutionary relationships at the gene level.

As shown in Figure 1, a species tree was reconstructed based on lefaxin gene sequences, revealing the phylogenetic relationships among *H. manillensis*, *W. pigra*, *H. nipponia*, and *H. medicinalis*. The branch model analysis revealed that all six lefaxin genes exhibited low ω values (≤0.08985), suggesting strong purifying selection and a high degree of conservation. The branch-site analysis further showed that, among the six lefaxin genes, *lefaxin_Hman2* had only 3.0776% of its sites under purifying selection, with the majority of sites exhibiting neutral selection. In contrast, the other five lefaxin genes exhibited 86.746–95.156% of their sites under purifying selection (*ω* ≤ 0.04615), with minimal neutral selection (*ω* = 1). The Bayes Empirical Bayes analysis identified several codon sites with potential positive selection (Table 2).

The site model analysis (M0, M7, M8) corroborated these results. The M0 model yielded an average ω of 0.06006, reflecting overall purifying selection. Models M7 and M8 confirmed that most sites were under purifying selection (*ω* = 0.00011–0.25513), with negligible positive selection (*ω* = 81.13, probability = 0.00001).

In summary, lefaxins were highly conserved, with most sites under strong purifying selection, highlighting their functional significance. The molecular evolution of lefaxins in *W. pigra* and *H. manillensis* was similar, dominated by purifying selection, likely due to their essential physiological roles in leeches.

### 3.3. Gene Expression

The mean TPM values ± standard deviations for the lefaxin genes are presented in Table 3. The total expression levels of the lefaxin genes in *H. manillensis* were 10,013.0 ± 5930.2, whereas in *W. pigra*, they were 29,041.4 ± 13,030.8. The total expression of the lefaxin genes was significantly higher in *W. pigra* than in *H. manillensis* (*Z* = −3.814, *p* < 0.001).

In *H. manillensis*, the Friedman test revealed a highly significant overall difference in the expression levels of the three lefaxin genes (Chi-Square = 51.063, *df* = 2, *p* < 0.0001). The Wilcoxon test showed significant differences in expression levels between all pairwise gene comparisons (*Z* < −2.281, *p* < 0.023). In *W. pigra*, the Friedman test also showed a highly significant overall difference in the expression levels of the three lefaxin genes (Chi-Square = 70, *df* = 2, *p* < 0.001). The Wilcoxon test showed significant differences in expression levels across all pairwise gene comparisons (*Z* < −5.159, *p* < 0.001).

A Mann–Whitney U test showed no significant difference in the expression levels of *lefaxin_Hman1* and *lefaxin_Wpig1*. However, highly significant differences in expression levels were observed between *lefaxin_Hman2* and *lefaxin_Wpig2*, as well as between *lefaxin_Hman3* and *lefaxin_Wpig3* (Table 3).

### 3.4. Protein Biochemical Properties

The sequence lengths of the homologous lefaxin genes were identical in *H. manillensis* and *W. pigra*. However, the molecular weights of lefaxins in *W. pigra* were generally slightly higher than those in *H. manillensis*. The pI values of the two species were not significantly different (*p* = 0.567). They were both below the physiological pH of mammals (7.35–7.45), suggesting that lefaxins carried a net positive charge under physiological conditions. The II values, which range from 0 to 40 for stable proteins, fell between 27.27 and 38.34 for lefaxins, indicating overall stability. Notably, lefaxins in *H. manillensis* exhibited slightly lower II than those in *W. pigra*, suggesting greater structural stability. The aliphatic indices ranged from 68.25 to 80.32. The aliphatic index reflects the volume occupied by aliphatic side chains in a protein, and higher values are commonly linked to enhanced thermal stability. *H. manillensis* showed marginally higher values, indicating enhanced thermal stability [57]. The GRAVY scores of all lefaxins were negative (−0.524 to −0.658), reflecting a general tendency to interact with water molecules. A more negative GRAVY score indicates greater hydrophilicity of the protein. Lefaxins in *W. pigra* exhibited slightly higher hydrophilicity values than those in *H. manillensis* (Table 4).

Differences in molecular weight, stability, and hydrophilicity between the two species may have reflected adaptive divergence in response to their ecological niches. *H. manillensis* lefaxins appeared to be more thermally and structurally stable, whereas *W. pigra* lefaxins displayed greater hydrophilicity and solubility, which may have supported their species-specific physiological functions.

### 3.5. Protein Structure Alignment

Structural alignment using the FATCAT algorithm revealed significant similarity between the archetypal lefaxin and six variants from *H. manillensis* and *W. pigra*, with all *p*-values ≤ 0.005. FATCAT scores ranged from 106.50 to 107.63, and RMSD values from 1.75 Å to 1.86 Å, reflecting minimal structural variation across 56 aligned residues. Notably, lefaxin_Hman2 and lefaxin_Wpig2 exhibited the highest FATCAT scores (107.63 and 107.57), while lefaxin_Wpig1 displayed the lowest RMSD (1.75 Å). These results suggested that the archetypal lefaxin exhibited substantial structural similarity with all six lefaxins, potentially supporting conserved biological functions at the molecular level.

### 3.6. Protein Docking

We performed protein–protein docking between the archetypal lefaxin and Factor Xa using ZDOCK and visualized the interacting residues with PyMOL. We then selected the identical residues from the archetypal complex as reference points and used them to compare the docking results of the six lefaxin variants (Table 5). The docking analysis revealed that many of these key residues were consistently involved across all six proteins, indicating that they played a crucial role in binding to Factor Xa and contributed to the stability of the docking interaction.

We evaluated docking quality using ZDOCK scores, where higher values indicated more stable interactions and lower energy conformations. Lefaxin_Hman2 and lefaxin_Wpig2 obtained relatively higher scores. Overall, the lefaxins from *W. pigra* showed slightly higher docking scores than those from *H. manillensis*, implying that they may have possessed greater structural stability or stronger affinity for protein–protein interactions.

### 3.7. Recombinant Protein Synthesis

**Figure 2:**
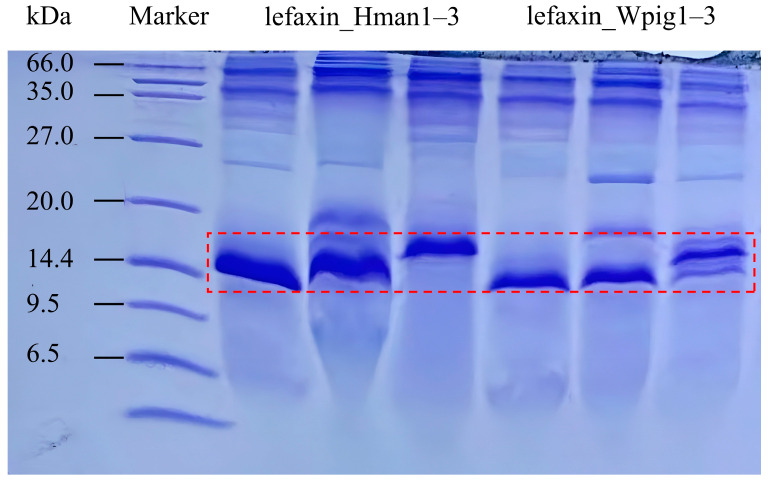
SDS-PAGE analysis of recombinant lefaxins. Lanes: M, protein marker; 1–6: lefaxin_Hman1, lefaxin_Hman2, lefaxin_Hman3, lefaxin_Wpig1, lefaxin_Wpig2, lefaxin_Wpig3. All recombinant proteins appeared as bands between 14.4 and 9.5 kDa, consistent with predicted molecular weights based on sequence length. Red dashed box indicates expected size range of target proteins.

We successfully expressed six recombinant lefaxins (lefaxin_Hman1–3 and lefaxin_Wpig1–3) in *Pichia pastoris*. After purification, the protein concentrations were 13.73, 30.40, 24.20, 25.44, 28.06, and 22.62 mg/mL, respectively. We predicted their molecular weights to be 13.86, 13.77, and 14.80 kDa for lefaxin_Hman1–3, and 13.90, 13.86, and 14.95 kDa for lefaxin_Wpig1–3. SDS-PAGE analysis confirmed that all recombinant proteins appeared at their expected molecular weights (Figure 2).

### 3.8. Anticoagulation of Lefaxins

To evaluate the anticoagulant properties of recombinant lefaxins, we conducted three sets of qualitative and three sets of quantitative replicate experiments, including all six target proteins from *H. manillensis* (lefaxin_Hman1–3) and *W. pigra* (lefaxin_Wpig1–3). Each assay was performed in three biological and three technical replicates. Anticoagulation performance was assessed using three clinical indicators: ACT, CR, and PF.

**Figure 3:**
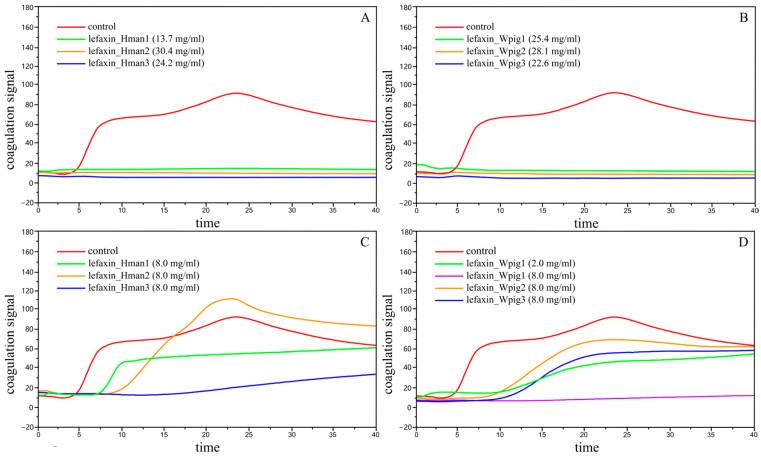
Coagulation signals of the control and recombinant lefaxins. (**A**) Coagulation curves for *H. manillensis* lefaxins (lefaxin_Hman1–lefaxin_Hman3) at their initial concentrations (13.7–30.4 mg/mL). (**B**) Coagulation curves for *W. pigra* lefaxins (lefaxin_Wpig1–3) at their initial concentrations (22.6–28.1 mg/mL). (**C**) Coagulation curves for *H. manillensis* lefaxins (lefaxin_Hman1–3) at a uniform concentration of 8.0 mg/mL. (**D**) Coagulation curves for *W. pigra* lefaxins (lefaxin_Wpig1–3) at 8.0 mg/mL, and for lefaxin1_Wpig1 at a reduced concentration of 2.0 mg/mL. The y-axis indicates coagulation signal intensity, and the x-axis represents time (min).

In qualitative tests, the parameters for the control group remained within the physiological range, confirming normal coagulation function. In contrast, all target proteins at their original concentrations resulted in complete suppression of coagulation, rendering ACT, CR, and PF values unmeasurable (Table 6). As shown in Figure 3A,B, the coagulation signal of the control group increased sharply after approximately 3 min, peaked, and then declined, indicating successful clot formation. However, the lefaxin-treated groups maintained flat signal curves, indicating vigorous anticoagulant activity.

For quantitative analysis, all lefaxins were tested at a standardized concentration of 8 mg/mL. As shown in Table 6 and Figure 3C,D, lefaxins from *W. pigra* exhibited markedly stronger anticoagulant activity than those from *H. manillensis*. In particular, lefaxin_Wpig1 completely suppressed coagulation at 8 mg/mL, with no measurable coagulation parameters due to full inhibition. Remarkably, even when diluted to 2 mg/mL, lefaxin_Wpig1 still maintained substantial anticoagulant activity, surpassing the performance of other proteins tested at 8 mg/mL.

In summary, lefaxin_Wpig1 exhibited the most potent anticoagulant effect among all tested proteins, and lefaxins from *W. pigra* overall displayed more vigorous anticoagulant activity than those from *H. manillensis*.

## 4. Discussion

This study compared the lefaxin family in two Asian leech species, *H. manillensis* and *W. pigra*, using next-generation sequencing techniques and recombinant expression assays. Molecular evolutionary analysis indicated that the *dN/dS* ratios for all lefaxins were significantly below 1 in both species, suggesting that these proteins were under strong purifying selection and tended to be conserved during evolution. Specific sites under neutral or positive selection indicated potential functional diversification. Further analysis revealed that *H. manillensis* lefaxins exhibited higher numbers of gene variant sites at both the DNA and protein levels, as well as greater haplotype numbers and a higher Watterson’s Theta diversity index compared to *W. pigra*. This divergence likely reflects the ecological habits and physiological traits of *H. manillensis*. As a tropical species, *H. manillensis* inhabits environments with higher ambient temperatures. As a poikilotherm, it has a relatively high metabolic rate [58]. The elevated metabolic rate likely increases the genomic mutation rate [59], thereby contributing to the higher genetic diversity observed in the lefaxin genes of *H. manillensis*.

Differences in lefaxin gene expression and anticoagulant activity between the two species suggest distinct evolutionary trajectories and molecular adaptation strategies. The elevated lefaxin expression in *W. pigra* may be phylogenetically attributed to its closer affinity to hematophagous leech genera, implying a blood-feeding ancestral lineage reliant on lefaxin-mediated anticoagulant activity. Although *W. pigra* has evolved into a non-bloodsucking species, the expression pattern of the lefaxin gene retains its ancestral characteristics due to its high conservation and short functional degradation.

Structural biology studies help explain why *W. pigra* lefaxins are better at preventing blood clots due to their similar structure, strong binding ability, and important interactions between specific parts of the molecules. Structurally, *W. pigra* lefaxins exhibit a high degree of similarity to the archetypal lefaxin, serving as a conserved scaffold that facilitates recognizing and binding to specific coagulation Factor Xa regions. Binding affinity determines the strength of their interaction [60], with *W. pigra* lefaxins demonstrating significantly higher affinity than other homologs, indicating their enhanced capacity to form stable complexes with Factor Xa. Key residue interactions mediate the anticoagulant function, stabilizing lefaxin–Factor Xa binding through multiple non-covalent bonds [61]. Critical binding sites in *W. pigra* lefaxin (e.g., K96 and Y99) are highly conserved relative to the archetypal lefaxin, correlating with its markedly superior anticoagulant activity compared to other homologs. These three factors act synergistically: structural similarity provides an optimal framework for residue functionality, while residue interactions reinforce binding affinity. Collectively, this molecular interplay confers high anticoagulant efficacy on the *W. pigra* lefaxins (especially lefaxin_Wpig1), effectively inhibiting blood coagulation in vitro.

In contrast, the low expression of lefaxins in *H. manillensis* may be due to a functional reallocation of anticoagulation strategies. As a hematophagous species, *H. manillensis* depends on blood ingestion for nutrient acquisition, necessitating rapid and potent anticoagulant mechanisms to sustain its feeding behavior. This species likely relies primarily on specialized anticoagulant proteins (e.g., hirudin) for immediate hemostatic suppression, thereby reducing selective pressure on lefaxin expression. The interplay of ancestral phylogenetic constraints and lineage-specific functional adaptations shapes the divergence observed between the two species. Specifically, *W. pigra* retains molecular traits constrained by its ancestral genetic architecture, whereas *H. manillensis* has undergone evolutionary specialization to prioritize functional efficiency in blood-feeding.

The non-hematophagous nature of *W. pigra* has long been a subject of skepticism within TCM, since its inclusion in the *Chinese Pharmacopoeia* [62]. According to TCM theory, leeches that suck blood are believed to possess special healing properties due to their bloodsucking behavior. Bloodsucking leeches, such as *H. manillensis* and *H. nipponia*, are thought to contain more active substances that help prevent blood clots, making them highly valuable in medicine. This study found that *W. pigra* does not rely on bloodsucking for sustenance, but its lefaxin proteins exhibit more vigorous anticoagulant activity. In contrast, *H. manillensis*, a hematophagous leech, exhibits weaker anticoagulant activity. This finding provides new scientific evidence supporting the medicinal value of *W. pigra*. It may alter the TCM perception of its efficacy, prompting the TCM community to reevaluate the criteria and theoretical basis for using leeches in medicine.

In molecular evolution, it is widely accepted that genes with more critical functions evolve more slowly than those with less important functions [63]. This principle is based on the neutral theory, which suggests a negative correlation between the functional importance of a gene and its rate of evolution. Specifically, molecules or segments with less functional importance tend to evolve more rapidly than those with more significant functional importance [64]. Since the anticoagulant function of lefaxins is vital for the survival of leeches, its sequence and structure undergo purifying selection during evolution, resulting in fewer variations. This evolutionary pattern is consistent with that of many other conserved genes. Lefaxin is more stable than other anticoagulant proteins, such as hirudin, and its well-preserved sequence suggests it has a reliable function in the organism [65]. Therefore, lefaxin may be an ideal target for the development of novel anticoagulant drugs.

From a pharmacological perspective, lefaxin offers potential advantages over classical anticoagulants such as hirudin. While hirudin acts as a direct thrombin inhibitor, lefaxin targets the upstream activator Factor Xa, thereby indirectly modulating thrombin generation. This mechanism may provide a more controllable anticoagulant effect and potentially reduce the risk of bleeding complications [66]. The evolutionary stability, structural conservation, and demonstrated anticoagulant activity of lefaxin underscore its promise as a novel target for the development of anticoagulant drugs.

This study closely compared the lefaxin family in two types of leeches, showing that lefaxins in *W. pigra* have more vigorous anticoagulant activity. This finding not only provides a scientific basis for the medicinal value of *W. pigra* but also has the potential to alter TCM’s perception of the efficacy of leeches. Lefaxin shows promise as a novel target for anticoagulant drugs. Future research could further explore the structure–function relationship of lefaxin, combined with drug design and optimization, to potentially develop more efficient anticoagulant drugs. Studying how lefaxin has evolved and been selected by different leech species will enhance our overall understanding of its development and function, providing new insights and opportunities for utilizing leech resources.

## 5. Conclusions

This study reveals that the non-hematophagous leech *W. pigra* exhibits more vigorous anticoagulant activity via its lefaxin proteins than the hematophagous *H. manillensis*. The findings challenge traditional assumptions about the medicinal efficacy of leech species and highlight lefaxins as a promising therapeutic target for thrombotic diseases. The research highlights the importance of reevaluating leech species for medicinal applications, providing a scientific basis for the inclusion of *W. pigra* in the development of anticoagulant drugs and expanding the potential of leech-derived biomolecules in modern medicine.

## Figures and Tables

**Table 1:** Intraspecific genetic variants of lefaxin genes and their encoded proteins.

Gene	Sequence Length	Coding Sequence			Protein Sequence		
		VS	HN	WD	VS	HN	WD
*lefaxin_Hman1*	363	4	5	0.00529	0	1	0.00000
*lefaxin_Hman2*	363	3	5	0.00397	1	2	0.00826
*lefaxin_Hman3*	378	7	10	0.00655	2	3	0.01058
*lefaxin_Wpig1*	363	3	5	0.00397	1	2	0.00826
*lefaxin_Wpig2*	363	2	3	0.00367	1	2	0.00826
*lefaxin_Wpig3*	378	1	2	0.00265	0	1	0.00000
Total	2208	20	30	—	5	11	—

Note: VS, number of variable sites; HN, number of haplotypes; WD, Watterson’s Theta diversity.

**Table 2:** Molecular evolutionary analysis of lefaxin genes under different selection models.

Gene	Branch Model	Branch-Site Model	Bayesian Empirical Bayesian Analysis
	Foreground *ω*	Site Class, Proportion, Foreground *ω*	Positive Sites (Probability. *ω* > 1)
*lefaxin_Hman1*	0.02863	0, 0.95156, 0.04615 1, 0.04844, 1.00000 2a, 0.00000, 1.00000 2b, 0.00000, 1.00000	41 T, 0.569
*lefaxin_Hman2*	0.01793	0, 0.03077, 0.04615 1, 0.00157, 1.00000 2a, 0.92080, 1.00000 2b, 0.04687, 1.00000	—
*lefaxin_Hman3*	0.08985	0, 0.86746, 0.03985 1, 0.04522, 1.00000 2a, 0.08299, 1.00000 2b, 0.00433, 1.00000	80 H, 0.557 81 A, 0.590
*lefaxin_Wpig1*	0.12575	0, 0.88938, 0.04210 1, 0.04605, 1.00000 2a, 0.06139, 1.00000 2b, 0.00318, 1.00000	27 N, 0.518 30 K, 0.551 61 E, 0.507
*lefaxin_Wpig2*	0.08171	0, 0.94221, 0.04591 1, 0.04738, 1.00000 2a, 0.00991, 1.00000 2b, 0.00050, 1.00000	87 K, 0.666
*lefaxin_Wpig3*	0.0592	0, 0.95156, 0.04615 1, 0.04844, 1.00000 2a, 0.00000, 1.00000 2b, 0.00000, 1.00000	48 T, 0.504

**Table 3:** Statistical analysis of lefaxin gene expression in *H. manillensis* and the *W. pigra*.

Gene	TPM (Mean ± SD)		Mann–Whitney U Test	
	*H. manillensis*	*W. pigra*	*Z*	*p*
*lefaxin1*	5394.3 ± 2064.9 ^a^	5729.7 ± 2729.1 ^b^	−0.402	0.688
*lefaxin2*	4540.1 ± 3822.4 ^a^	23,205.8 ± 10,273.6 ^a^	−6.803	<0.001
*lefaxin3*	78.6 ± 42.9 ^b^	105.9 ± 28.1 ^c^	−3.289	<0.001
total	10,013.0 ± 5930.2	29,041.4 ± 13,030.8	−3.814	<0.001

Note: Different superscript letters show statistically significant difference between TPM values of different genes; *lefaxin1*–*3* indicate *lefaxin_Hman1–3* or *lefaxin_Wpig1*–*3*.

**Table 4:** Basic biochemical properties of the lefaxin proteins.

Protein	Sequence Length	Molecular Weight (Da)	pI	II	Aliphatic Index	GRAVY
lefaxin_Hman1	120	13,856.54	5.64	29.55	76.33	−0.524
lefaxin_Hman2	120	13,772.50	5.69	27.27	69.08	−0.588
lefaxin_Hman3	125	14,800.67	5.75	34.55	80.32	−0.626
lefaxin_Wpig1	120	13,904.55	5.77	34.05	73.08	−0.600
lefaxin_Wpig2	120	13,858.55	5.69	27.79	68.25	−0.631
lefaxin_Wpig3	125	14,947.90	5.72	38.34	78.00	−0.658

Note: pI, isoelectric point; II, instability index; GRAVY, grand average of hydropathicity.

**Table 5:** Protein–protein docking residue interactions and ZDOCK scores between Factor Xa and six lefaxin variants from *H. manillensis* and *W. pigra*.

Protein	Interacting Residue Pairs Between Factor Xa and Each Lefaxin	ZDOCK Scores
lefaxin_Hman1	G2-G216, N107-G218, M1-D189	881.685
lefaxin_Hman2	S70-Q192, S70-R143, S68-G218, S68-G216, L120-Q61, K66-K96, K66-Y99	1028.977
lefaxin_Hman3	R41-G218, D45-K96	669.794
lefaxin_Wpig1	E103-K96, E103-Y99, G2-G216, K110-R222	932.989
lefaxin_Wpig2	M1-G218, M1-D189, M1-A190, T112-N107, T112-R222	1123.996
lefaxin_Wpig3	G114-S173, M1-S195, H3-Y99, I5-K96, N2-D102, R4-I175	955.779

**Table 6:** Anticoagulant activities (mean ± SD) of recombinant lefaxins at different concentrations.

Treat	ACT	CR	PF
Control	228.3 ± 14.7	23.6 ± 1.9	2.5 ± 0.4
lefaxin_Hman1 (13.7 mg/mL)	*∞*	*∞*	*∞*
lefaxin_Hman2 (30.4 mg/mL)	*∞*	*∞*	*∞*
lefaxin_Hman3 (24.2 mg/mL)	*∞*	*∞*	*∞*
lefaxin_Wpig1 (25.4 mg/mL)	*∞*	*∞*	*∞*
lefaxin_Wpig2 (28.1 mg/mL)	*∞*	*∞*	*∞*
lefaxin_Wpig3 (22.6 mg/mL)	*∞*	*∞*	*∞*
lefaxin_Hman1 (8.0 mg/mL)	371.7 ± 35.5	16.6 ± 2.4	0.3 ± 0.0
lefaxin_Hman2 (8.0 mg/mL)	453.7 ± 93.1	13.0 ± 1.7	1.3 ± 0.5
lefaxin_Hman3 (8.0 mg/mL)	863.7 ± 344.2	4.2 ± 2.2	0.3 ± 0.0
lefaxin_Wpig1 (8.0 mg/mL)	*∞*	*∞*	*∞*
lefaxin_Wpig1 (2.0 mg/mL)	547.3 ± 80.7	6.4 ± 2.4	0.3 ± 0.0
lefaxin_Wpig2 (8.0 mg/mL)	539.0 ± 46.8	8.0 ± 0.3	0.3 ± 0.0
lefaxin_Wpig3 (8.0 mg/mL)	690.3 ± 124.6	6.6 ± 2.1	0.3 ± 0.0

Note: ACT, activated clotting time; CR, clot rate; PF, platelet function; “∞” indicates complete inhibition of coagulation, with no detectable clotting signal.

## Data Availability

Data are contained within the article and Supplementary Files.

## Supplementary Materials

The following supporting information can be downloaded at https://www.mdpi.com/article/10.3390/biology14080918/s1. File S1: Coding and amino acid sequences of lefaxins from *H. manillensis*, *W. pigra*, *H. nipponia*, and *H. medicinalis*; File S2: lefaxin coding sequences from 32 *H. manillensis* samples; File S3: lefaxin coding sequences from 35 *W. pigra* samples.

## Abbreviations

TCM	traditional Chinese medicine
VS	number of variable sites
HN	number of haplotypes
WD	Watterson’s Theta diversity
TPM	transcripts per million
pI	isoelectric point
II	instability index
GRAVY	grand average of hydropathy
RMSD	root mean square deviation
YEPD	Yeast Extract Peptone Dextrose
ACT	activated clotting time
CR	clot rate
PF	platelet function
